# Genome-wide identification, classification and expression analysis of *MYB* gene family in coconut (*Cocos nucifera* L.)

**DOI:** 10.3389/fpls.2023.1263595

**Published:** 2024-01-15

**Authors:** Jing Li, Shukuan Guo, Yin Min Htwe, Xiwei Sun, Lixia Zhou, Fangyuan Wang, Chunru Zeng, Shuangyan Chen, Amjad Iqbal, Yaodong Yang

**Affiliations:** ^1^ Coconut Research Institute, Chinese Academy of Tropical Agricultural Sciences/Hainan Key Laboratory of Tropical Oil Crops Biology, Wenchang, Hainan, China; ^2^ School of Tropical Crops, Yunnan Agricultural University, Kunming, Yunnan, China; ^3^ Department of Food Science & Technology, Abdul Wali Khan University Mardan, Mardan, Pakistan

**Keywords:** MYBs, genome-wide analysis, color, RNA-seq, qPCR, coconut

## Abstract

MYB transcription factors regulate the growth, development, and secondary metabolism of plant species. To investigate the origin of color variations in coconut pericarp, we identified and analyzed the *MYB* gene family present in coconut. According to the sequence of *MYB* genes in Arabidopsis thaliana, homologous *MYB* gene sequences were found in the whole genome database of coconut, the conserved sequence motifs within *MYB* proteins were analyzed by Motif Elicitation (MEME) tool, and the sequences without conservative structure were eliminated. Additionally, we employed RNA-seq technology to generate gene expression signatures of the R2R3-*MYB* genes across distinctive coconut parts exhibiting diverse colors. To validate these profiles, we conducted quantitative PCR (qPCR). Through comprehensive genome-wide screening, we successfully identified a collection of 179 *MYB* genes in coconut. Subsequent phylogenetic analysis categorized these 179 coconut *MYB* genes into 4-subfamilies: 124 R2R3-*MYB*, 4 3R-*MYB* types, 4 4R-*MYB* type, and 47 unknown types. Furthermore, these genes were further divided into 34 subgroups, with 28 of these subgroups successfully classified into known subfamilies found in Arabidopsis thaliana. By mapping the *CnMYB* genes onto the 16 chromosomes of the coconut genome, we unveiled a collinearity association between them. Moreover, a preservation of gene structure and motif distribution was observed across the *CnMYB* genes. Our research encompassed a thorough investigation of the R2R3-*MYB* genes present in the coconut genome, including the chromosomal localization, gene assembly, conserved regions, phylogenetic associations, and promoter cis-acting elements of the studied genes. Our findings revealed a collection of 12 R2R3-MYB candidate genes, namely *CnMYB8*, *CnMYB15*, *CnMYB27*, *CnMYB28*, *CnMYB61*, *CnMYB63*, *CnMYB68*, *CnMYB94*, *CnMYB101*, *CnMYB150*, *CnMYB153*, and *CnMYB164*. These genes showed differential expressions in diverse tissues and developmental stages of four coconut species, such as *CnMYB68*, *CnMYB101*, and *CnMYB28* exhibited high expression in majority of tissues and coconut species, while *CnMYB94* and *CnMYB164* showed lower expression. These findings shed light on the crucial functional divergence of *CnMYB* genes across various coconut tissues, suggesting these genes as promising candidate genes for facilitating color development in this important crop.

## Introduction

All eukaryotic organisms contain the *MYB* transcription factor (TF) family, which is known for its extensive size and its ability to regulate a wide range of physiological processes in plants. These processes include environmental adaptation, hormone signal transduction, development, metabolic regulation, (Li et al., 2019; Li T. et al., 2023; Ma et al., 2023). The name *MYB* is derived from the conserved DNA-binding domain known as the *MYB* domain. The MYB proteins can be classified into different groups based on the repetitive units they possess: 1R-MYB (1- repeat), R2R3-MYB (2-repeats), R1R2R3-MYB (3-repeats), and 4R-MYB (4-repeats). Each repeat contains approximately 50-53 amino acids and encodes three α-helices, with the second and third helices forming a helix-turn-helix (HTH) structure (Du et al., 2012). 1R-MYB plays an important role in regulating plant transcription and maintaining chromosome structure. The R2R3-MYB family genes contain two conserved R2 and R3 repeat sequences in the MYB binding domain, as well as a regulatory domain (activation or inhibition function) in the C-terminus variable region. It has numerous members and diverse functions, widely participating in cell differentiation, secondary metabolism, environmental stress, and invasion of diseases and pests; The conserved domains of the 3R-MYB family genes are composed of R1, R2, and R3, which are mainly involved in the regulation of cell differentiation and cell cycle; The conserved domain of the 4R-MYB subfamily genes consists of four R1/R2 repeat sequences. The plant TF database (http://planttfdb.gaolab.org) contains a total of 22,032 MYB and 15,369 MYB-related sequences (Tian et al., 2020). The MYB family is abundant in numerous plant species, with specific examples including 197 *AtMYB* genes in *Arabidopsis thaliana* (Katiyar et al., 2012), 155 *PaMYB* genes in *Petunia axillaris* (Chen et al., 2021), 174 *MrMYB* genes in *Myrica rubra* (Cao et al., 2021), 133 *DcMYB* genes in *Dendrobium catenatum* (Zhang et al., 2021), and 159 *EgMYB* genes in *Elaeis guineensis* (Zhou et al., 2020). The R2R3-MYB subfamily is the most prevalent among plants, distinguished by the presence of two types of R domains located at the N-terminal end. These R2R3-MYB proteins commonly demonstrate transcriptional activation or repression capacity at their C-terminal end (Tolosa and Zhang, 2020). Because of these DNA-binding domain characteristics, the R2R3-MYB family was also divided into 25 subgroups in *Arabidopsis* (Stracke et al., 2001). In other plant species, the R2R3-MYB proteins can be classified into 25 or more classes (Li et al., 2020; Ding et al., 2023).

Pigments are significant secondary metabolites that play vital roles in photosynthesis and petal coloration. Extensive research has shown that *MYB* genes are involved in pigment formation within various plant parts, such as petals and peels (Chen et al., 2021; Yang et al., 2021). Anthocyanins are natural, water soluble pigments that play a vital role in plants. They contribute a variety of colors to reproductive organs and vegetative tissues, ranging principally from reddish to purplish or bluish shades (Li et al., 2022). The *MYB* genes in plants play a crucial role in the accumulation of anthocyanins and have a significant influence on the development of color in various organs such as floral parts, leaves, and pericarp (Mackon et al., 2021). Numerous studies have established the crucial role of R2R3-*MYB* transcription factor in directlyre gulating genes expression associated with anthocyanin biosynthesis. It serves as a key regulator in controlling anthocyanin production in various horticultural plants, including vegetables, fruits, and ornamentals (Duan et al., 2022; Wang et al., 2022; Jiang et al., 2023). The *AaMYB2*, an R2R3*-MYB* gene isolated by Li et al. (2016) from *Anthurium andraeanum* (Hort.), has been identified as a specific transcriptional regulator for anthocyanin production in spathes and leaves. Furthermore, recent studies have revealed that certain members of the R2R3*-MYB* family also act as principal regulators of carotenoid synthesis (Sagawa et al., 2016; Zhu et al., 2017; Ampomah-Dwamena et al., 2019; Li et al., 2020; Yin et al., 2022). Thus, the influence of R2R3*-MYB* genes extends beyond anthocyanin synthesis and encompasses the regulation of carotenoid production as well.

**Figure 1:**
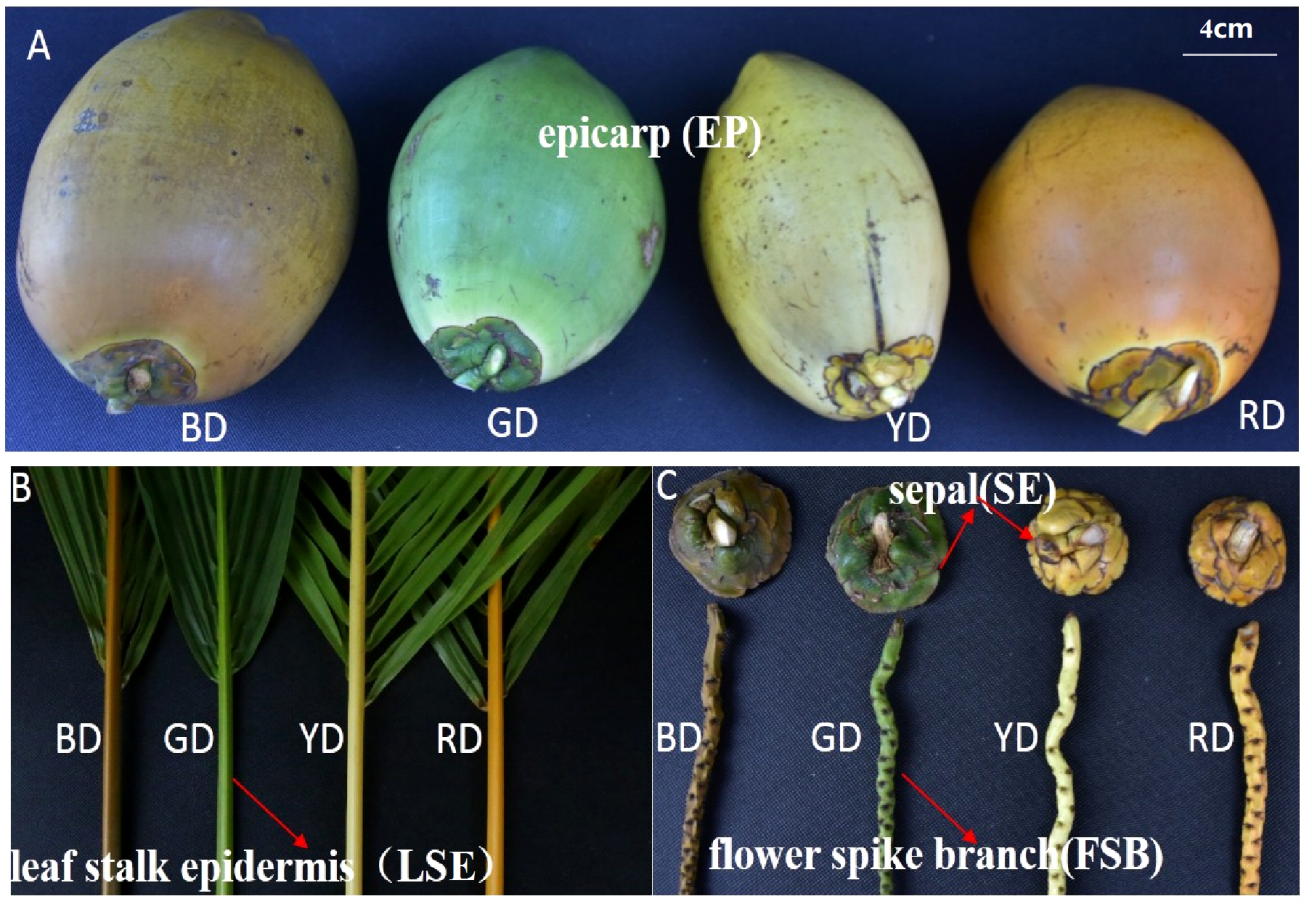
Different tissue parts of different coconut species. Leaf stalk epidermis (LSE), epicarp (EP), sepal (SE) and flower spike branch (FSB) of 7-month-old fruit of Red dwarf Coconut (RD), Yellow dwarf Coconut (YD), Brown dwarf Coconut (BD) and Green dwarf Coconut (GD).

Coconut (*Cocos nucifera* L.) is an important fruit tree and woody oil crop that thrives in hot regions and possesses a unique quality trait- the color of its epicarp. Coconut peels can display a range of colors, including orange, brown, yellow, and green. Interestingly, the leaf stalk epidermis (LSE), sepal (SE), and flower spike branch (FSB) of different coconut species exhibit the same color as their respective peels (**Figure 1**). In contrast to many other fruits, the outer skin color of the coconut remains relatively stable throughout its development, without undergoing significant changes. Unlike some fruits that undergo a distinct transition process associated with maturity, coconut do not exhibit such a marked transformation. Availability of the comprehensive genome sequence of the coconut (Xiao et al., 2017) has provided a valuable resource for analyzing *MYB* genes across the entire genome.

Although several studies have established the role of *MYB*transcription factors (TFs) in many biochemical and physiological processes in plants, a comprehensive identification and characterization of *MYB* TFs specific to coconuts is still lacking. Additionally, the expression patterns of *MYB* genes in different parts of the coconut are not yet fully understood. Notably, there has been no comprehensive systematic analysis of the entire array of *MYB* genes conducted in coconuts. Therefore, addressing this knowledge gap and conducting a comprehensive genomic analysis of the *MYB* gene family, with a specific emphasis on R2R3-*MYB* in coconuts, is crucial. The information provided by these results may help further analyze the function of the CnMYB gene and elucidate its secondary metabolic mechanism. This report aims to address this critical need by focusing on comprehensive genomic profiling and expression analysis of *MYB* genes in coconuts, with a specific emphasis on highlighting the R2R3-*MYB* subgroup.

## Materials and methods

### Identification of *CnMYB* genes

The draft coconut genome served as the reference for obtaining the coconut MYB gene sequences (Xiao et al., 2017). A Hidden Markov Model (HMM) profile representing the MYB DNA-binding domain with accession (PF00249) was acquired from the Pfam protein family database (http://pfam.xfam.org/) (Finn et al., 2016). Subsequently, this profile was employed as a query (P < 0.001) for the identification of all potential *CnMYB* genes. In parallel, *AtMYB* gene sequences were utilized as query sequences to detect MYB genes within the coconut genome. The identified *MYB* genes containing conserved domains underwent further analysis, while those lacking the PF00249 conserved domain were excluded from the dataset. Amino acid sequences of *Arabidopsis* MYB proteins (AtMYBs) were obtained from the *Arabidopsis* Information Resource (TAIR) database (https://www.arabidopsis.org/). The BLAST searches were conducted against the coconut genome databases using *Arabidopsis* MYB protein sequences as queries to identify *CnMYB* gene families (Katiyar et al., 2012). In total, 179 *CnMYB* genes were identified from the coconut genome. Additional information regarding the number of amino acids, molecular weight (MW) and isoelectric point (pI) of each MYB protein of interest was gathered using the ExPASy proteomic website (https://web.expasy.org/compute_pi/). The CELLO tool was used to predict the intracellular distribution of all *CnMYB* genes.

### Comprehensive analysis of coconut *MYB* genes

A neighbor-joining (NJ) phylogenetic analysis was conducted by MEGA-X based on the alignment. Bootstrap analysis with 1000 replicates was performed to calculate the reliability of the NJ tree (Kumar et al., 2018). The necessary data, including mRNA sequences, CDS and gene annotation summaries of coconut *MYB* genes, were retrieved from Gigascience Database 2017 (http://dx.doi.org/10.5524/100347) (**Supplementary Table 1**). To confirm the structures of the coconut *MYB* genes, the mRNA sequences were aligned with the complete coconut genome sequence. The Gene Structure Display Server was then employed to analyze and determine the intron-exon organization of the coconut *MYB* genes. For the analysis of conserved sequence motifs within MYB proteins, the Motif Elicitation (MEME) tool (http://meme-suite.org/tools/meme) (Bailey et al., 2009) was utilized, and the results are presented in **Supplementary Table 2**. Additionally, the TBtools software (version 1.045) developed by Chen et al. (2020) was used to visualize the chromosomal localization of *MYB* genes in the coconut genome. This analysis utilized the annotated genomic data from the coconut genome database to accurately map the *MYB* genes onto the corresponding chromosomes.

### Plant materials

Samples from four distinct species of coconuts, namely Red Dwarf (RD), Yellow Dwarf (YD), Brown Dwarf (BD), and Green Dwarf (GD), were carefully selected for analysis. The specific parts examined included the leaf stalk epidermis (LSE), epicarp (EP), sepal (SE), and flower spike branch (FSB) of 7-month-old fruits (**Figure 1**). Nine coconut fruits from the same fruit bunch were taken. Three coconut fruits were taken for collection of peel, sepals, floral branches and petioles separately and mixed them to form one biological replicate. In this way two different group were prepared to make another two biological replicate. These research samples were generously provided by the Coconut Research Institute (CRI), Chinese Academy of Tropical Agricultural Sciences (CATAS), located in Wenchang, Hainan, China. Following collection, all samples were immediately cryopreserved using liquid nitrogen and subsequently transferred to a freezer set at -80°C for future use.

### RNA-seq analysis

The RNA extraction was carried out by the MRIP method (Method for RNA isolation from Palm) (Xiao et al., 2012) and has been improved. The protocol of the RNA extraction method according to the Iqbal et al. (2019). The quality of the extracted RNA (i.e., degradation and contamination) was assessed using 1% agarose gels. Additionally, the integrity of the RNA was determined using the Agilent 2100 Bioanalyzer (Agilent Technologies, CA, United States), while the concentration was measured using a Nanodrop Spectrophotometer (IMPLEN, CA, United States). Sequencing of the samples was performed on the BGISEQ-MGI2000 platform instrument at BGI Genomics (Shenzhen 518083, China) with three biological replicates for each sample. To ensure high-quality data, the raw sequencing reads were processed to obtain clean reads. This involved excluding reads with adapters, reads with unknown bases more than 5% and low-quality base ratios more than 20% were filtered using SOAPnuke (version 1.4.0) (Chen et al., 2018). The resulting clean reads were stored in FASTQ format. Subsequently, the data were aligned to the reference genome using HISAT (v2.1.0) (Kim et al., 2015), and then matched with the assembled unique genes using Bowtie2 (v2.2.5) (Langmead and Salzberg, 2012). RNA-seq by expectation maximization (RSEM) (version 1.2.8) was utilized to calculate the expression levels of the genes (Li and Dewey, 2011). For functional annotation, the assembled unigenes were annotated using databases such as KEGG and GO, and transcription factors were predicted as well. Differential gene analysis within groups was conducted using DESeq with the conditions of Fold Change ≥ 2 and adjusted P-value ≤ 0.001 (Wang et al., 2010).

### Analysis of MYB gene expression in coconut using transcriptome data

To conduct a comprehensive analysis of coconut *MYB* genes, transcriptoe datasets from various coconut tissues, including LSE, EP, SE and FSB, were utilized. The expression levels of genes were quantified using Reads Per Kilobase Million (RPKM) values, which were further Log2 transformed to facilitate comparative analysis. To visualize the expression patterns of 12 R2R3-*MYB* genes, a heatmap was generated using TBtools.

### RNA isolation and quantitative PCR (qPCR) analysis

Forward and reverse primers for qPCR analysis were designed by National Center for Biotechnology Information (NCBI) Primer-BLAST (http://www.ncbi.nlm.nih.gov/tools/primer-blast) with melting temperatures of 55-60°C, primer length 19-25bp, GC content 50-60% and amplicon size of 80-200bp. To prevent amplification of non-target gDNA, the primers were designed to span intronic regions. The properties of each primer were evaluated using the PCR Primer Stats software. The primers used for qPCR analysis can be found in **Supplementary Table 4**. The reference gene CnACTIN was used as an internal control (Xia et al., 2014).The Quick and Reliable RNA Extraction Method (QRREM) was employed to isolate total RNA from the epicarp of coconut fruit, following the protocol of Iqbal et al. (2019). Subsequently, the isolated RNA underwent quality and quantity assessment through agarose gel electrophoresis and Nanodrop spectrophotometer analysis. For reverse transcription, 1 μg of RNA was used with the MightyScript first-strand cDNA synthesis kit following the manufacturer’s instructions. The qPCR reactions were conducted using the 2 × SYBR Green qPCR ProMix in 96-well optical plates on a Mastercycler ep *realplex^4^* machine. The qPCR reactions were carried out with a total reaction volume of 10 μL, consisting of amplification at 95°C for 5 s, 55°C for 15 s, and 68°C for 20 s. The melting stage involved heating from 60°C to 95°C for 20 min. Each experiment was conducted with biological and technical triplicates. The fold change in expression level for each sample was calculated by normalizing the CT value relative to a reference gene, using the 2^-ΔΔCt^ method (Livak and Schmittgen, 2001).

### Data analysis

The experiments were conducted in triplicate (n=3) to ensure the reliability of the findings. Mean values, accompanied by standard errors of the mean, were used to present the data. SAS software (SAS Inc., Cary, NC, USA) was employed to perform ANOVA (analysis of variance) and DMRT (Duncan’s Test) with a significance level set at p < 0.05 and p < 0.01 to assess the significance among various treatments. Correlation coefficients were calculated based on the mean values.

## Results

### Genomic-scale profiling of MYB genes in coconut

A comprehensive analysis of *MYB* genes in the coconut genome revealed a total of 179 *MYB* genes, which wereeffectively profiled from the coconut genome. According to the numbering in the coconut genome, it is arranged from small to large and positioned as *CnMYB01* to *CnMYB179*. Detailed information about each of these profiled *MYB* genes as shown in **Supplementary Tables 1** and **2**. The length of the peptide chains in the CnMYB proteins varied from 61 to 1150 amino acids, as shown in **Supplementary Table 1**. Additionally, the predicted proteins exhibited a molecular weight range of 7.8 to 128.1 and isoelectric points ranging from 4.09 to 10.11, as indicated in **Supplementary Table 1**. Regarding the intracellular distribution of the CnMYB putative proteins the vast majority was predicted to be localized in the nucleus. Based on the analysis of the N-terminal aa region, 154 CnMYBs were predicted to be localized in the nucleus whereas CnMYB02 would be localized in the cytoplasm and CnMYB03 and CnMYB132 both in the cytoplasm and nucleus (**Supplementary Table 1**).

### Genomic localization of *CnMYB* genes

**Figure 2:**
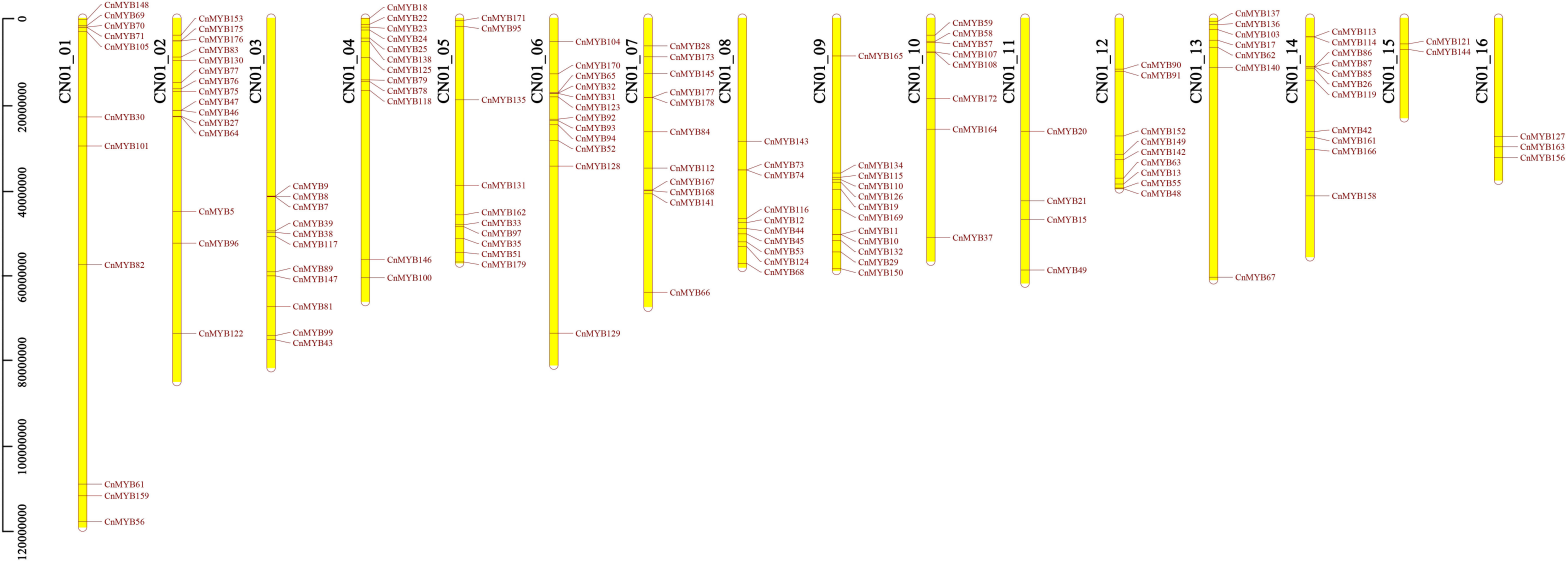
*CnMYB* genes distribution across 16 chromosomes of coconut genome. The scale represents the length of coconut chromosomes.

Through a search using DNA sequence annotations, we observed that 179 identified coconut *MYB* genes were distributed across 16 chromosomes. Among these genes, 148 genes exhibited uneven distribution patterns across the chromosomes (**Figure 2**). More specifically, our analysis revealed that 11 *CnMYB* genes were located on chromosome 1, 15 genes on chromosome 2, 11 genes on chromosome 3, 12 genes on chromosome 4, 10 genes on chromosome 5, 12 genes on chromosome 6, 11 genes on chromosome 7, 10 genes on chromosome 8, 12 on chromosome 9 held, 8 genes on chromosome 10 held, 4 genes on chromosome 11, 9 genes on chromosome 12, 13 genes on chromosome 13, 11 genes on chromosome 14, 2 genes on chromosome 15, and 3 genes on chromosome 16 (**Figure 2**). Additionally, 29 *CnMYB* genes were assigned to chromosomes that could not be determined. Notably, chromosome 2 contained the highest number of *CnMYB* genes (15), while chromosome 4, 6 and 9 each contain 12 *CnMYB* genes. In contrast, chromosome 15 had the lowest number of MYB genes (2) (**Figure 2**).

### Exon-intron organization and motif patterns of *CnMYB* genes

**Figure 3:**
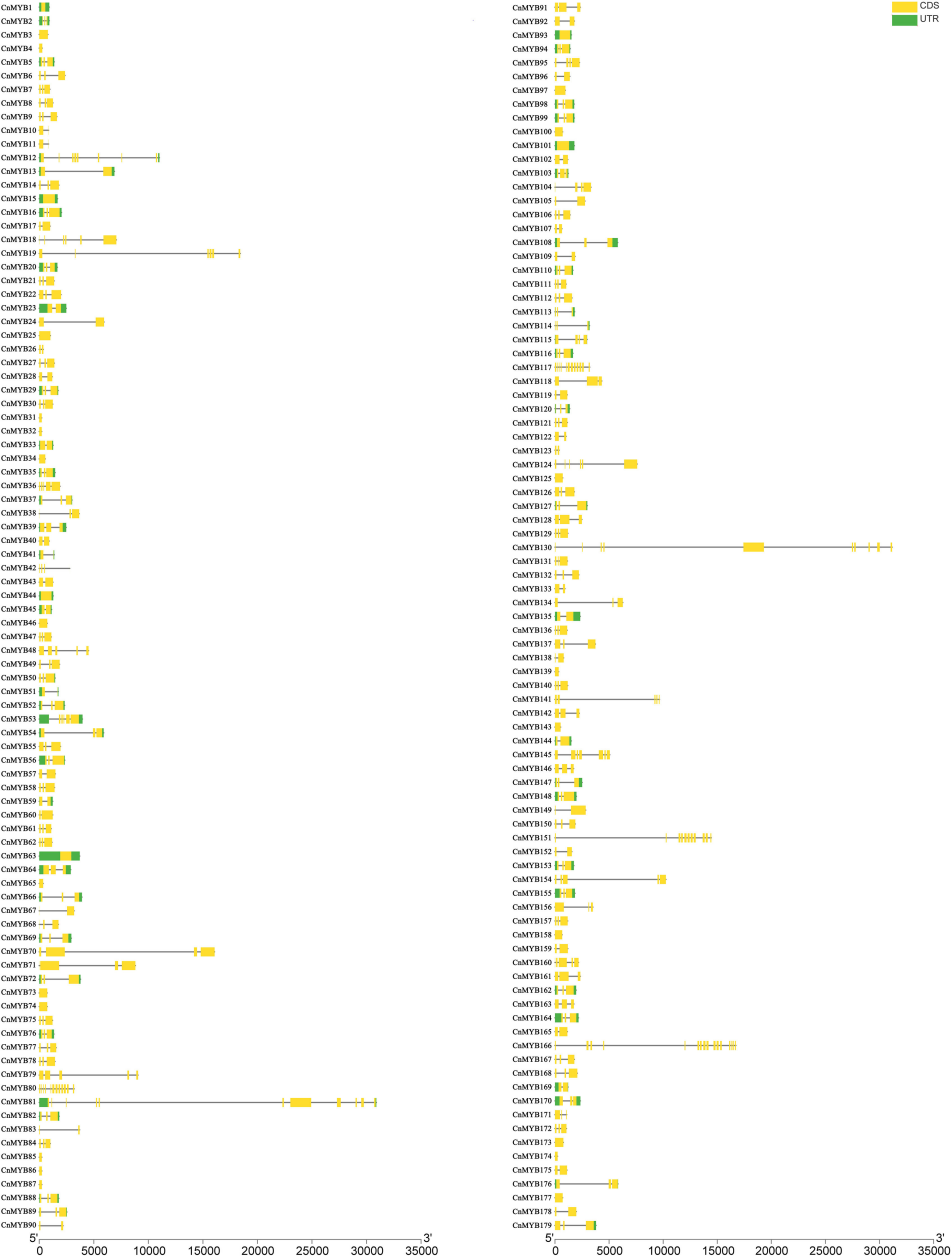
Gene structure of 179 *MYB* genes from Coconut.

**Figure 4:**
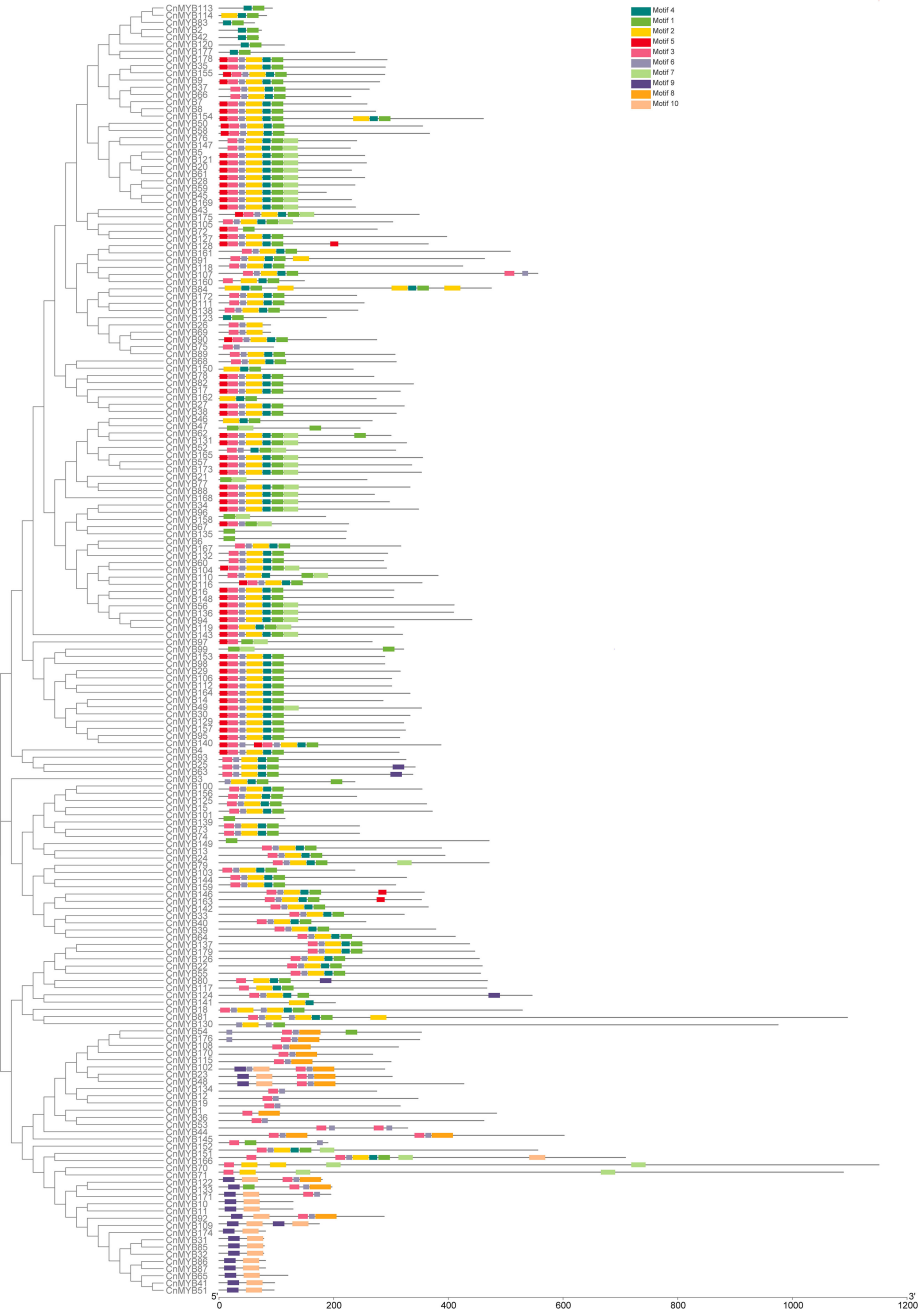
Conserved motif of the CnMYB proteins. All motifs were identified by MEME.

**Figure 5:**
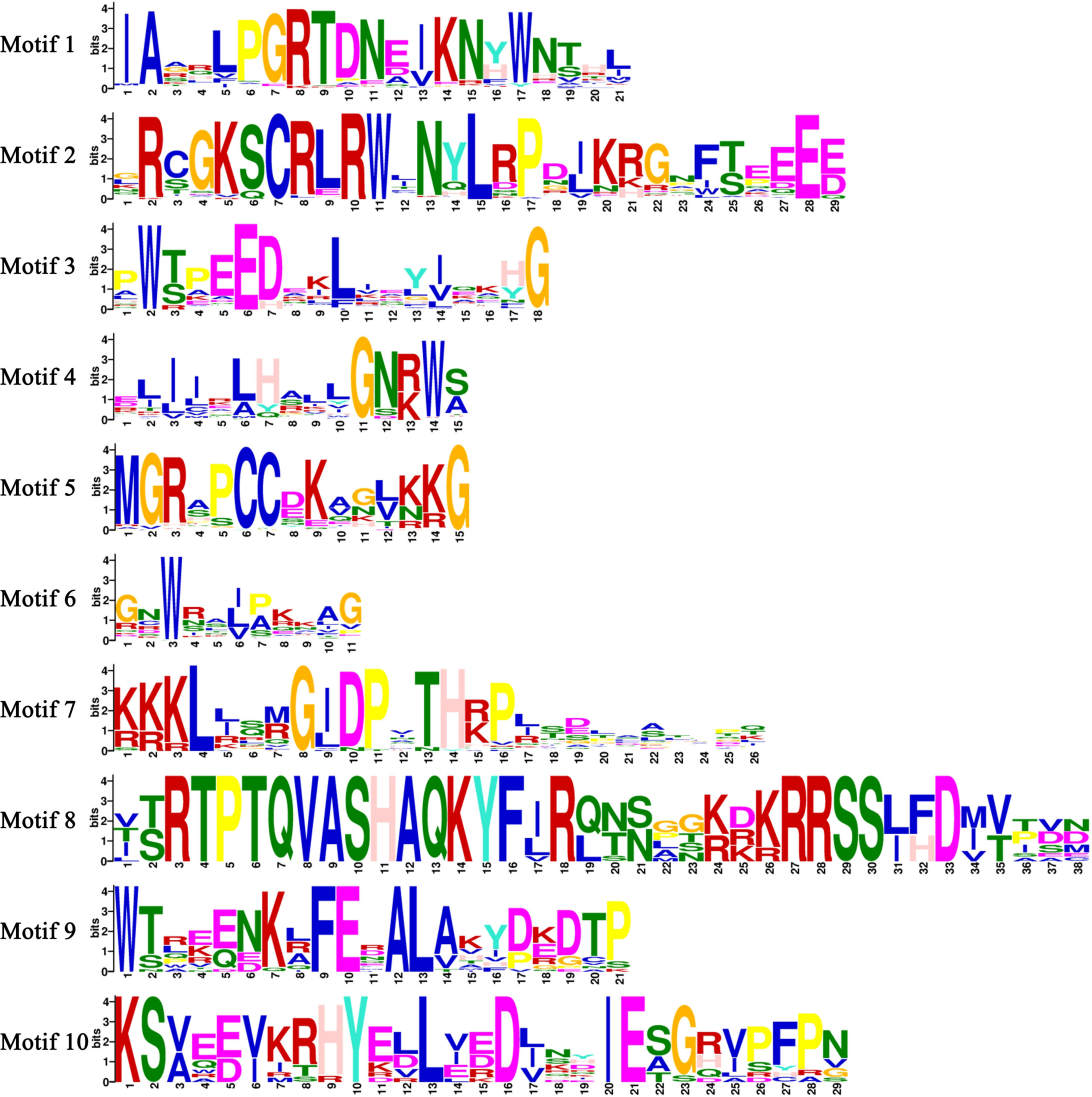
A total of ten conserved motifs distribution in *CnMYB* genes. Each motif is represented by a number (1-10) in the colored box. Sequence logos of amino acid residues of 10 conserved motifs of CnMYB proteins are also shown.

The exon-intron organization of the 179 *CnMYB* genes was examined using the Gene Structure Display Server program, as illustrated in **Figure 3**. The analysis revealed that the majority *MYB* genes exhibited varying numbers of introns, ranging from 0 to 15 (**Figure 3**). Among the 179 *CnMYB* genes, *CnMYB166* had the highest number of introns (15), followed by *CnMYB80* and *CnMYB117* with 11 introns each. Additionally, *CnMYB81* and *CnMYB151* contained 10 introns, while *CnMYB130* had 9 introns. Furthermore, *CnMYB12* had 7 introns, and *CnMYB145* had 6 introns. Notably, 28 genes, including *CnMYB1*, *CnMYB3*, *CnMYB4*, *CnMYB15*, *CnMYB25*, *CnMYB31*, *CnMYB32*, *CnMYB34*, *CnMYB44*, *CnMYB46*, *CnMYB63*, *CnMYB65*, *CnMYB73*, *CnMYB74*, *CnMYB85*, *CnMYB86*, *CnMYB87*, *CnMYB93*, *CnMYB97*, *CnMYB100*, *CnMYB101*, *CnMYB125*, *CnMYB139*, *CnMYB143*, *CnMYB158*, *CnMYB173*, *CnMYB174*, and *CnMYB177*, did not contain any introns. Furthermore, three genes (*CnMYB80*, *CnMYB117* and *CnMYB166*) had the maximum number of exons (≥12). Most of the *MYB* genes exhibited a small number of introns, typically ranging from 0 to 3, suggesting a conserved pattern of intron distribution within the *MYB* genes. The disparity observed in the *CnMYB* gene composition suggests noteworthy deviation within the coconut genome. Additionally, we performed protein motif profiling to assess the variation in the *CnMYB* gene family of coconut. Our findings revealed the presence of 10 preserved motifs across all 179 identified MYB genes, as depicted in **Figures 4**, **5** and **Supplementary Table 3**.

### Phylogenetic analysis of *CnMYB* genes

**Figure 6:**
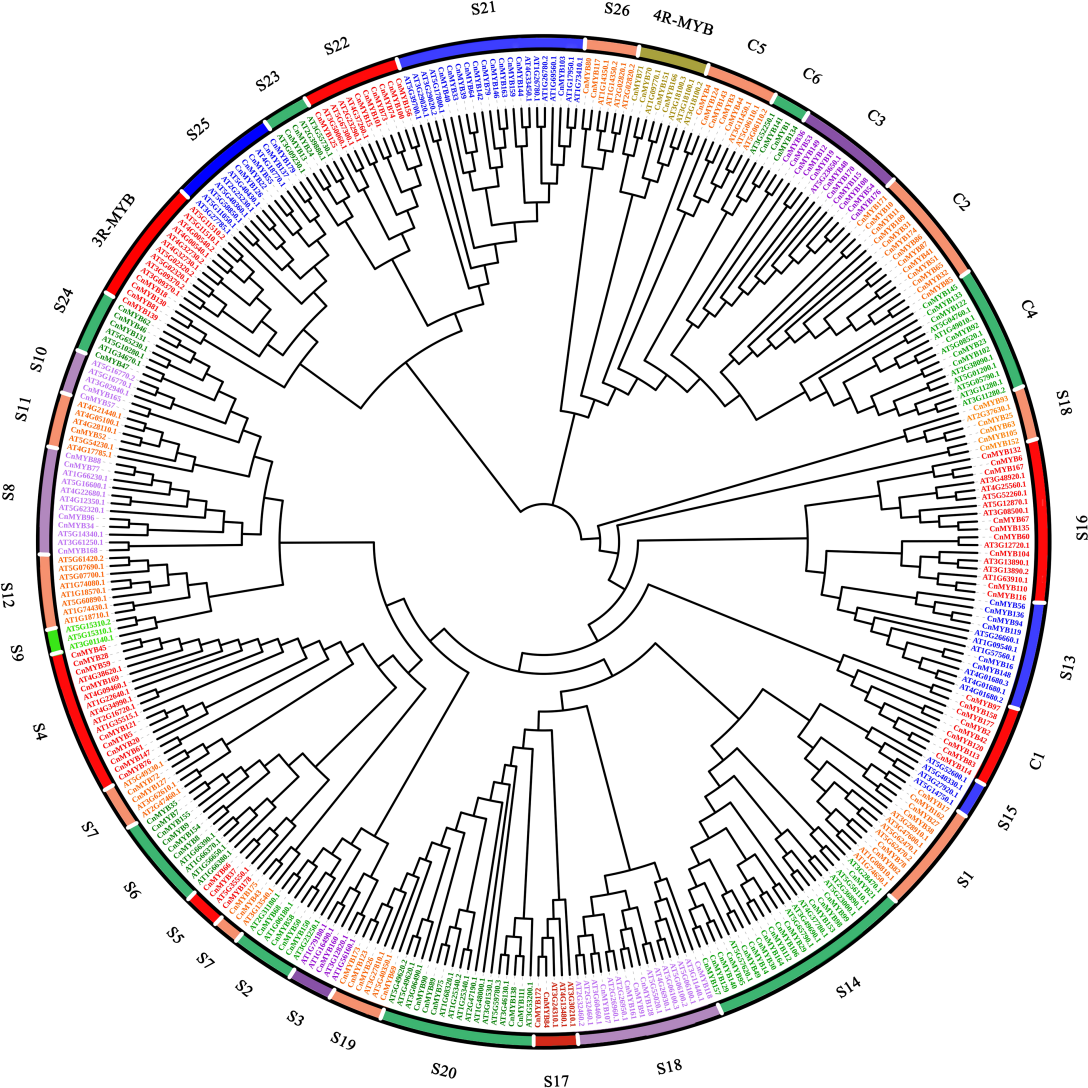
Phylogenetic analysis of 179 *CnMYB* (coconut) and 130 *AtMYB* (*Arabidopsis*) genes. A maximum likelihood (ML) phylogenetic tree of 309 *MYB* genes of two plants was constructed using MEGA 6.06 software with protein sequences.

To construct a phylogeny of MYB proteins, a maximum likelihood (ML) method was employed, using 179 MYB proteins sequences from coconut and 154 MYB proteins sequences from *Arabidopsis thaliana* (**Figure 6**). The resulting dendrogram classified the *MYB* genes into 34 distinct subgroups, denoted as S1-S26, C1-C6, 3R-*MYB*, and 4R-*MYB* (**Figure 6**). These subgroups represent four main types of MYB protein families: 4R-MYB, 3R-MYB, R2R3-MYB, and the coconut-specific subgroups (**Figure 6**). Out of the 34 subgroups, 29 included proteins from both coconut and *Arabidopsis*, while the remaining five were specific to either coconut (S9, S12 and S15) or *Arabidopsis* (C1 and C2). Similar species-specific subgroups of MYBs have been found in other plant species, such as *Solanum tuberosum* (Li Y. et al., 2020), *Casuarina equisetifolia* (Wang et al., 2021) and *Petunia* (Chen et al., 2021). Within the 3R*-MYB* subfamily, we identified four *CnMYB* genes (*CnMYB18*, *CnMYB81*, *CnMYB130*, *CnMYB139*) and ten *AtMYB* genes. Similarly, the 4R-*MYB* subfamily consisted of four *CnMYB* genes (*CnMYB70*, *CnMYB71*, *CnMYB151*, *CnMYB166*) and four *AtMYB* genes. The C1 to C6 subfamily included forty-seven *CnMYB* genes and thirteen *AtMYB* genes. The remaining 124 *CnMYB* genes and 127 *AtMYB* genes were associated with the R2R3-*MYB* family. The R2R3-*MYB* family was further divided into subgroups S1-S26, with varying numbers of *CnMYB* and *AtMYB* genes. Notably, subgroups S3 and S11 comprised only one *CnMYB* member each, making them the smallest groups, while subgroup S14 comprised 15 members, making it the largest group. Interestingly, no *CnMYB* genes were found in subgroups S9, S12, or S15, suggesting potential gene loss during coconut genome evolution or acquisition in Arabidopsis evolution. The presence of more *CnMYB* genes than *AtMYB* genes in certain subgroups indicates functional distinction of *MYB* genes among various plant species. These phylogenetic findings suggest that *MYB* genes clustered within the same set may share preserved functions, which should be further investigated through experimental approaches.

### Expression profiling of *CnR2R3-MYB* Genes in various tissues

**Figure 7:**
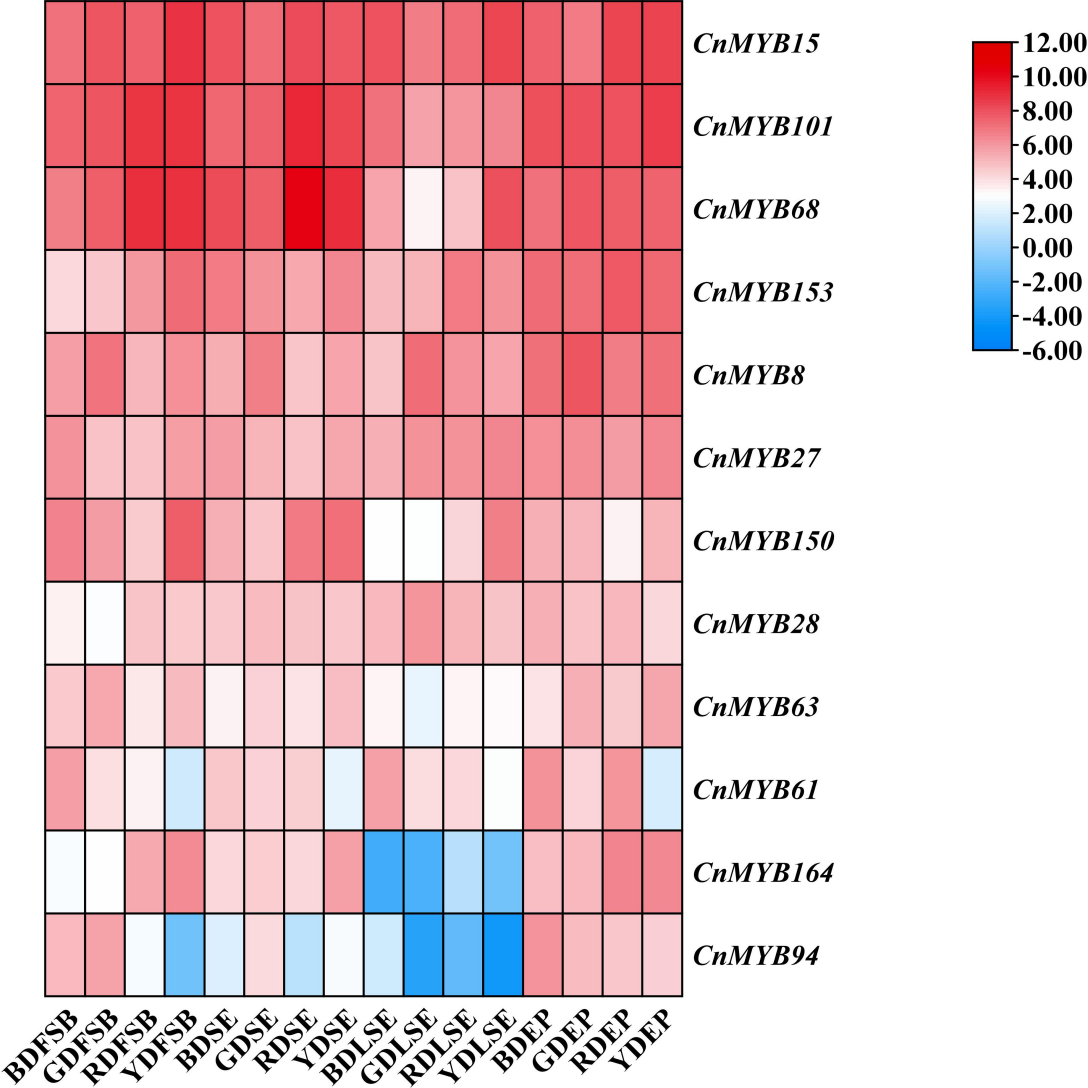
Heat map of candidate genes expression in different tissues of different coconut species. Note: Log_2_^FPKM^ value were sued to construct the heat map with clustering.

We conducted an analysis of the expression profiles of 179 *CnMYB* genes in four distinct tissues of coconut, including LSE, EP, SE and FSB using RNA-seq data from a database. The results were visualized using a heatmap, which revealed the expression patterns of 12 *R2R3-MYB* genes in coconut tissues (**Figure 7**, **Supplementary Table 5**). Among these genes, three (*CnMYB15*, *CnMYB68*, *CnMYB101*) exhibited the highest expression across all four coconut tissues (**Figure 7**). Conversely, genes with the lowest expression levels were predominantly observed in LSE, specifically *CnMYB94* and *CnMYB164* (**Figure 7**). Notably, genes with higher expression levels were primarily observed in the EP tissues of all four coconut species (**Figure 7**). Furthermore, the expression of *CnMYB8* was found to be higher in all four tissues of the Green Dwarf (GD) species compared to other coconut varieties.

### Expression analysis of candidate *CnMYB* genes during coconut fruit development

**Figure 8:**
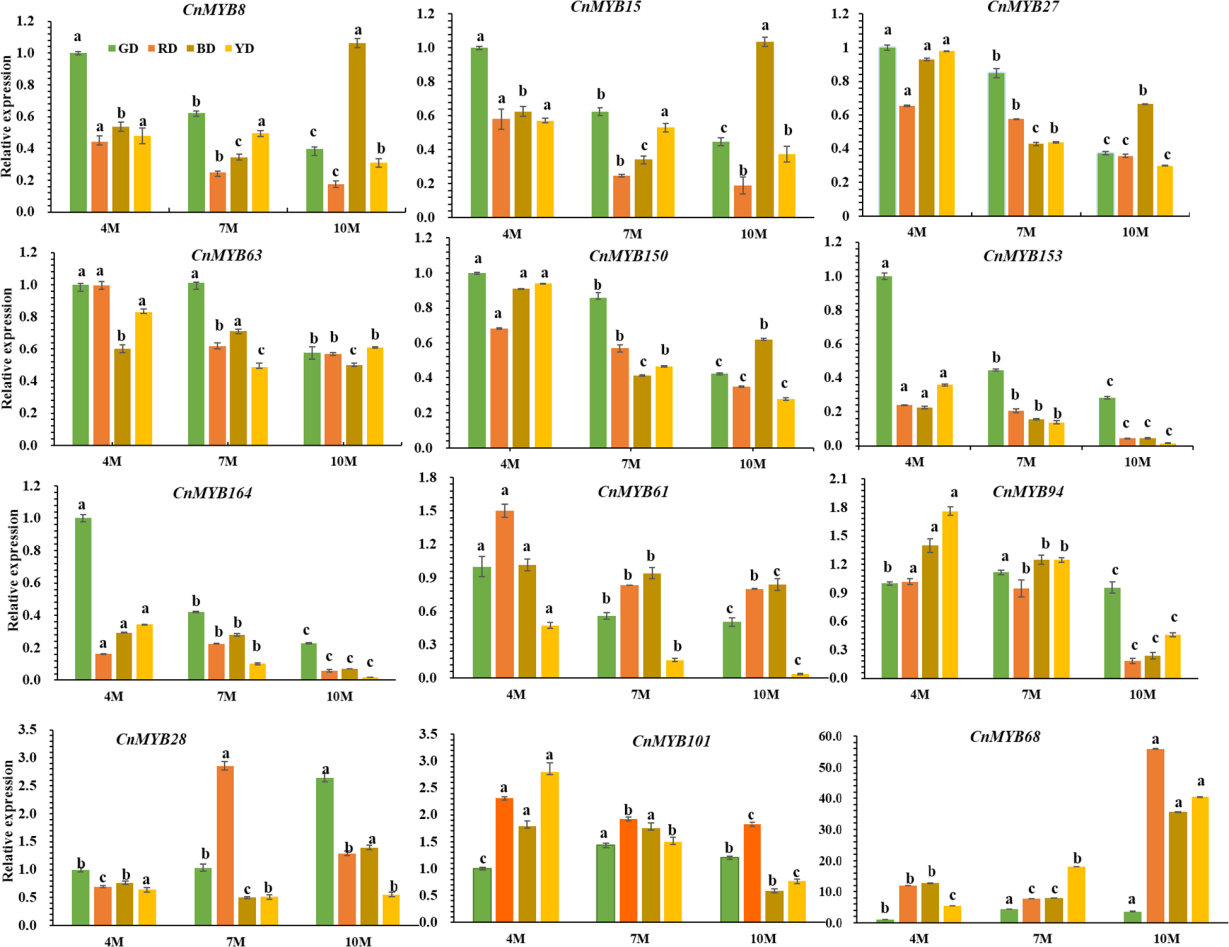
Relative expression levels of *CnMYB8*, *CnMYB15*, *CnMYB27*, *CnMYB28*, *CnMYB61*, *CnMYB63*, *CnMYB68*, *CnMYB94*, *CnMYB101*, *CnMYB150*, *CnMYB153*, *CnMYB164* genes in the peel of different coconut varieties at different developmental stages, using CnActin as an internal reference gene. Data represent the mean ± SD of three independent experiments. Different lowercase letters indicate significant differences according to the least significant difference test (LSD) at p < 0.05.

Moreover, we performed qPCR analysis to investigate the expression patterns of 12 candidate *MYB* genes (*CnMYB8*, *CnMYB15*, *CnMYB27*, *CnMYB28*, *CnMYB61*, *CnMYB63*, *CnMYB68*, *CnMYB94*, *CnMYB101*, *CnMYB150*, *CnMYB153*, *CnMYB164*) during different stages of fruit development (4M, 7M, and 10M). Among these genes, *CnMYB28*, *CnMYB68*, and *CnMYB101* displayed higher expression levels compared to the other genes, especially *CnMYB68* (**Figure 8**). Notably, the expression level of *CnMYB68* in the 10-month-old fruit peel of Red Dwarf (RD), Yellow Dwarf (YD), and Brown Dwarf (BD) coconuts was significantly higher than in other stages and Green Dwarf (GD) coconut (P<0.05). Additionally, the expression levels of *CnMYB101* in RD, YD, and BD were higher than in GD, with the highest expression level observed in the 4-month-old coconut peel of RD, YD, and BD (**Figure 8**). Conversely, the expressions of *CnMYB8*, *CnMYB15*, *CnMYB27*, *CnMYB63*, *CnMYB150*, *CnMYB153*, and *CnMYB164* were relatively lower compared to the other genes.

## Discussion

### Identification and phylogenetics of R2R3-MYB genes in coconut

MYB transcription factors have been extensively studied and recognized as one of the largest families of transcription factors in plants. Among all MYB subgroups, the R2R3-MYB protein comprises the highest number of members that can play a crucial role in various aspects of the secondary metabolism in plants (Song et al., 2021; Zhang et al., 2021). The R2R3-*MYB* family can control the diverse biological processes such as plant metabolism, growth and development (Li et al., 2023; Zhang et al., 2023). Additionally, it regulates plant responses to hormones and various types of stresses (Zhao et al., 2023). Several genomic-scale investigations have been concluded to identify *MYB* family members in various plant genomes (Li Y. et al., 2020; Liu et al., 2021; Zhao et al., 2023). The genomic identification of R2R3-MYB transcription factors has been conducted in various plant species, revealing their significant presence in plant genomes. For instance, extensive sequencing efforts have led to the identification of 138 *R2R3-AtMYBs* in *Arabidopsis thaliana* (Katiyar et al., 2012), 99 *R2R3-DcMYB* in *Dendrobium catenatum* (Zhang et al., 2021), 393 *R2R3-TaMYBs* in *Triticum aestivum* (Wei et al., 2020), 111 *R2R3-StMYB* in *Solanum tuberosum* (Li Y. et al., 2020) and 174 *R2R3-NtMYB* in *Nicotiana tabacum* (Yang et al., 2022). However, little is known about the *MYB* gene family in *Cocos nucifera*.

In this study, we identified 179 MYB members in *Cocos nucifera* through bioinformatics analysis, and characterized their phylogenetic relationships with A. thaliana MYBs. Our compresinsive analysis encompassed phylogenetic characteristics, physical and chemical properties, gene structure, chromosome location, tissue-specific expression patterns, and expression profiles during coconut fruit development stages. Our findings revealed that the MYB domain and other motifs of CnMYBs exhibit a high degree of conservation with those of *Arabidopsis* AtMYBs. Nonetheless, we also observed divergence between *CnMYBs* and *AtMYBs*, indicating a combination of conservation and diversity within plant MYBs.

Specifically focusing on the R2R3-MYB family, our analysis identified 124 members within the coconut genome. Phylogenetic analysis categorized these members into 26 subfamilies, with unified subfamily members displaying similar motifs and gene structures (**Figure 6**). The oil palm genome was found to contain 152 R2R3-MYB family members, which were classified into 23 subfamilies (Zhou et al., 2020). Furthermore, the protein sequences of the 124 coconut R2R3-MYB family members exhitited varying lengths, ranging from 89 (*CnMYB123*) to 555 (*CnMYB118*) amino acids, with an average length of 314 amino acids. Their molecular masses also varied, spanning from 10.37 (*CnMYB123*) to 59.79 kDa (*CnMYB118*), with an average molecular mass of 34.84 kDa. Theoretical isoelectric points ranged from 4.28 (*CnMYB90*) to 10.06 (*CnMYB107*), with an average molecular mass of 6.99 (**Supplementary Table 1**). In a separate investigation involving 124 longan R2R3-MYB family members, their protein sequences ranged from 134 to 661 amino acids, with an average length of 307 amino acids. The molecular masses ranged from 15.69 to 775.81 kDa, with an average of 34.68 kDa and theoretical isoelectric points ranged from 4.91 to 10.6 (Lv et al., 2023). Based on the findings of a previous study conducted by Zhang et al. 2021, our study provides further support for the hypothesis that the MYB gene family exhibits a significant level of evolutionary conservation among various plant species.

### Gene structure and protein motif analysis of R2R3-MYB genes in coconut

The pattern of gene structure is a useful tool for studying the evolutionary associations within a gene family. In our study, we identified 124 *R2R3-CnMYB* genes, which exhibited a range of exon numbers from 1 to 16 (**Figure 3**). Most of the R2R3-CnMYBs, similar to those found in other plant species, consisted of three exons and two introns (Liu et al., 2014; Chen et al., 2022; Du et al., 2022). It is worth noting that within the same subfamily, the exon/intron patterns of *R2R3- CnMYB* genes showed remarkable, with the majority of genes having no more than two introns. This finding aligns with previous research that also observed the presence of a maximum of two introns in most plant R2R3-MYB genes (Sabir et al., 2022; Yin et al., 2022). Furthermore, we analyzed the motif compositions of CnMYB proteins. Our findings revealed that the majority of MYB proteins contained motifs 1, 2, 3, 4, 5, and 6 (**Figure 4**), while most MYB proteins contained motifs 1, 3, 5, and 8 in *Dendrobium catenatum* (Zhang et al., 2021). The majority of MYB genes, included several motifs (1, 2, 3, 4, and 5) in *Prunus avium* (Sabir et al., 2022). These motifs were conserved within specific subgroups, and proteins in the same subgroup that share these motifs likely (Qin et al., 2023).

### R2R3-CnMYB genes may be involved in pigment formation of coconut epicarp

Prior research has implicated R2R3-MYB genes in essential functions in the anthocyanins, carotenoids and flavonoid biosynthesis in a variety of plants. For instance, in red-fleshed apple, *MdMYB22* and *MdMYB12* have been identified as key regulatorsof proanthocyanidins and flavonols biosynthesis (Wang et al., 2017). Moreover, *PpMYB10* has been found to primarily control anthocyanin biosynthesis in the exocarp of *Prunus persica* (Ravaglia et al., 2013), while *MdMYB1* is a major regulator of anthocyanin biosynthesis in red-skinned fruit (Zhang et al., 2019). In pear fruit, *PbMYB12b* has been found to positively regulate flavonol biosynthesis by enhancing the expression of *PbCHSb* and *PbFLS* (Zhai et al., 2019). In wolfberry, *Lba11g0183* and *Lba02g01219* have been identified as candidate genes involved in carotenoid biosynthesis (Yin et al., 2022). Additionally, in *Elaeis guineensis*, the *VIR* gene, encoding R2R3-MYB-like transcription factor with homology to Lilium LhMYB12 and similarity to Arabidopsis PRODUCTION OF ANTHOCYANIN PIGMENT1 (PAP1), regulate the heterogeneity of red and yellow fruit color (Singh et al., 2014). Genetic variations in the *VIRESCENS* gene have been associated with the conspicuousness of fruit colors in palms, indicating potential selection by frugivores (Wang et al., 2022). Notably, when comparing the *VIRESCENS* gene sequences of oil palm and date palms, no matching R2R3-*MYB* gene was found in coconut. In coconut, the *VIRESCENS* gene appears to be disrupted by the insertion of a highly repetitive sequence spanning 100 kb (Wang et al., 2022).

The influence of light exposure time on flavonoid and anthocyanin biosynthesis in fruits has been extensively studied by Premathilake et al. (2020). It was found that exposing pear fruit to light for a long duration up-regulated the expression of the R2R3-MYB DNA-binding protein *PpMYB17* (Premathilake et al., 2020), resulting in higher anthocyanin biosynthesis (Alabd et al., 2022). In our study, we observed that the epicarp covered by sepals exhibited a lighter or even whiter color, which may be attributed to the obstruction of other coconut fruits within the clusters, which can result in variations in the overall pericarp color. It is speculated that the uneven coloration is influenced by light obstruction (**Supplementary Figure 1**). We also identified higher expression levels of *CnMYB68*, *CnMYB101*, and *CnMYB28* in various tissues and developmental stages across the four coconut species, as evident from both transcriptome data and quantitative data. However, further experiments are required to confirm their functional roles.

## Conclusions

This study successfully identified 179 *MYB* genes in the coconut genome through a comprehensive genome-wide screening approach. A thorough investigation was conducted into the genomic architecture, genetic lineages, chromosomal localization, gene replication events, preserved motifs, and expression patterns across different tissues. The expression pattern analysis of *CnMYB*s in various coconut tissues revealed their constitutive expression with significant functional differentiation. Additionally, qPCR testing of 12 selected *CnMYB* genes demonstrated their diverse expression patterns. These findings provide valuable insights into the essential functional divergence observed among *CnMYB* genes across diverse coconut tissues, establishing them as potential candidate genes responsible for color development in this important crop. Furthermore, this research offers a comprehensive understanding of the *MYB* gene family in coconut, laying a strong foundation for future explorations into the functional roles and evolutionary dynamics of *MYB* genes in coconut.

## Data availability statement

The data presented in the study are deposited in the NCBI repository, accession number: PRJNA374600. The transcriptome data used in this article is attached in **Supplementary Table 5**.

## Author contributions

YY: Writing – review & editing. JL: Data curation, Writing – original draft, Writing – review & editing. SG: Data curation, Writing – review & editing. YMH: Writing – review & editing. XS: Writing – review & editing. LZ: Software, Writing – review & editing. FW: Writing – review & editing. CZ: Writing – review & editing. SC: Writing – review & editing. AI: Writing – review & editing.

## Supplementary material

Supplementary Figure 1 Coloring of different coconut varieties.
