# Supplementary figures and images for: Genome-wide identification, classification and expression analysis of MYB gene family in coconut (Cocos nucifera L.)
Source: Front Plant Sci. 2024 Jan 15;14:1263595. doi: 10.3389/fpls.2023.1263595 (PMC10822967; doi:10.3389/fpls.2023.1263595)

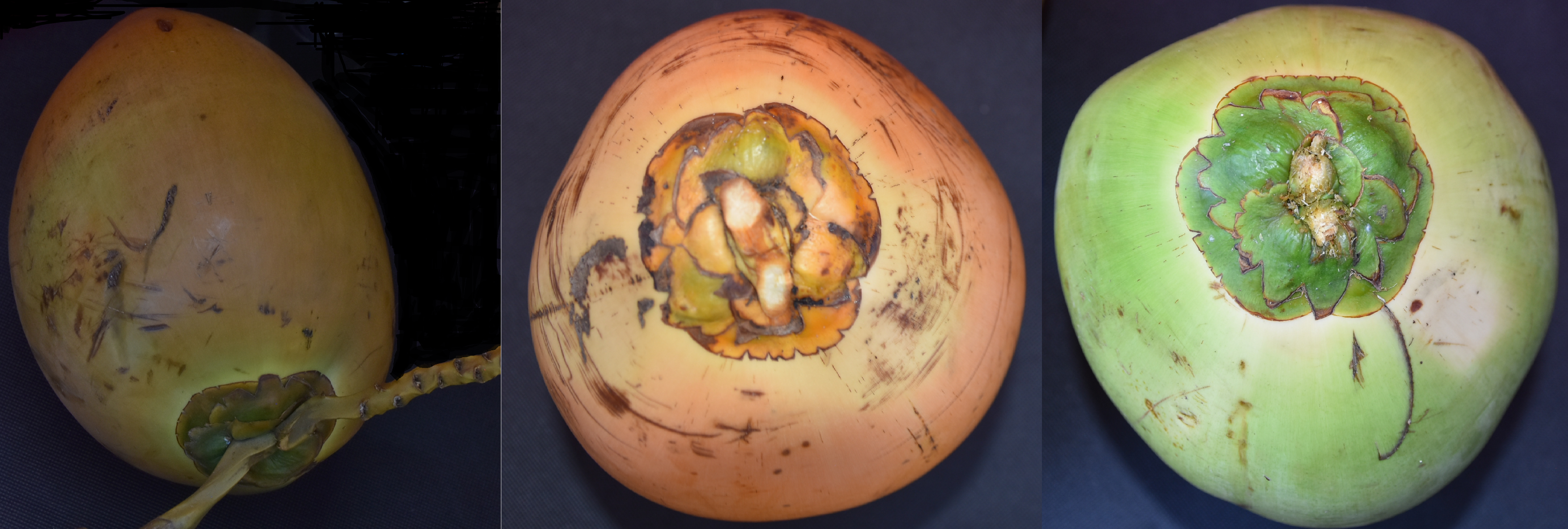

Supplement: Supplementary Figure 1 — Coloring of different coconut varieties. [file Image_1.tif]
